# On‐Chip Silicon‐Based Micro‐Reflector Pixelated with Micro‐Light‐Emitting Diodes for Augmented Reality Projection Displays

**DOI:** 10.1002/advs.202523254

**Published:** 2026-01-07

**Authors:** Wenzong Lai, Junhu Cai, Zhengui Fan, Yu Chen, Ziming Yao, Shuzhan Yan, Yun Ye, Sheng Xu, Qun Yan, Tailiang Guo, Enguo Chen

**Affiliations:** ^1^ National and Local United Engineering Laboratory of Flat Panel Display Technology College of Physics and Information Engineering Fuzhou University Fuzhou Fujian P. R. China; ^2^ Fujian Science & Technology Innovation Laboratory for Optoelectronic Information of China Fuzhou Fujian P. R. China

**Keywords:** augmented reality, beam shaping, micro‐reflector, projection engines, silicon‐based device, µLED

## Abstract

Augmented reality (AR) displays demand optical engines that are compact, efficient, and highly directional. While micro‐light‐emitting diodes (µLEDs) provide exceptional brightness, fast response, and low power consumption, their poor light extraction and near‐Lambertian emission severely limit optical coupling in AR systems. Here, we introduce a pixel‐by‐pixel on‐chip silicon‐based micro‐reflector device (SMRD) for µLEDs that simultaneously integrates light‐extraction enhancement and beam shaping within a scalable architecture. Simulation and experimental results show that the SMRD narrows the emission divergence from ±70.5° to ±39.4°, enhances luminous flux within ±20° by ∼64%, and suppresses pixel crosstalk to 4.75%. Beyond device‐level improvements, a near‐eye display prototype integrating the µLED/SMRD device with a projection lens, beam splitter, metal mask, and a camera acting as an eye substitute further verifies its system‐level performance. The SMRD thus delivers a compact and fabrication‐compatible route toward high‐efficiency, miniaturized AR projection engines. This work establishes a foundation for µLED‐based light‐field control, with implications for next‐generation near‐eye displays, spatial light modulators, and advanced photonic systems.

## Graphical abstract

This paper presents a pixel‐by‐pixel on‐chip silicon‐based micro‐reflector device (SMRD) for µLEDs that enables simultaneous light‐extraction enhancement and directional emission control. The scalable architecture significantly improves forward light utilization and suppresses pixel crosstalk. System‐level validation in a near‐eye display prototype highlights its potential for compact, high‐efficiency AR optical engines.

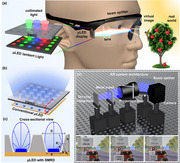

## 1 Introduction

Augmented reality (AR) has advanced rapidly in recent years, with applications spanning consumer electronics, industrial manufacturing, remote collaboration, and smart healthcare. Its primary goal is to seamlessly overlay virtual information onto the physical world, thereby enabling immersive human–machine interaction [1, 2, 3].

Mainstream AR systems typically consist of an optical projection engine and a combiner [4, 5], which can be realized through various approaches such as conventional optics [6, 7], waveguides [8, 9], retinal projection [10], and light‐field displays [11, 12]. Each approach presents inherent trade‐offs in display quality, efficiency, device compactness, and system complexity. For example, conventional optics deliver excellent image fidelity but are bulky; Waveguides offer compact designs and wide eyebox but suffer from low efficiency and high luminance requirements; Light‐field displays are structurally simple and efficient but prone to screen‐door artifacts; And retinal projection achieves high efficiency and direct imaging yet often depends on laser scanning, which complicates integration. Among these factors, brightness remains a critical bottleneck for AR systems [13]. Because virtual images are viewed through the combiner against ambient scenes, insufficient source luminance causes dim or indistinguishable images under bright conditions. Therefore, enhancing the effective eyebox luminance is a key requirement in AR optical engine design and device development.

To achieve sufficient eyebox luminance while maintaining system efficiency, AR optical engines must meet stringent demands for output power, response speed, and integration [14, 15, 16, 17]. Micro‐light‐emitting diodes (µLEDs), with their miniaturization, high brightness, fast response, and low power consumption, are widely regarded as promising light sources for next‐generation near‐eye displays [18, 19, 20]. Realizing adequate luminance, however, requires efficient management of the complete optical energy conversion chain, including internal quantum efficiency (IQE) [21, 22], light‐extraction efficiency (LEE) [23, 24, 25], collimation efficiency [26, 27, 28, 29], and transmission efficiency of subsequent optics [10]. In particular, the intrinsically low LEE and near‐Lambertian wide‐angle emission of µLEDs severely constrain optical coupling and light utilization in miniature optical systems, constituting a major bottleneck for AR performance enhancement [15, 30].

In recent years, significant efforts have been devoted to enhancing the efficiency and directionality of µLEDs. Internal approaches, including surface roughening [31, 32, 33, 34], sidewall passivation [35, 36], and photonic crystal structures [37, 38, 39, 40, 41], promote vertical light coupling. External strategies employ metasurfaces [42, 43, 44], resonant cavities [45, 46, 47, 48], and microlens arrays [15, 27, 49] to tailor emission profiles and improve directionality. Each method has distinct advantages and limitations. Photonic crystals and metasurfaces achieve excellent beam shaping and extraction enhancement but require costly, complex fabrication; Microlens arrays are fabrication‐ready and versatile but yield modest improvements; Resonant cavities enhance both extraction and directionality but pose design and processing challenges; Surface roughening or sidewall passivation provides simple, high‐gain improvements without precise directional control. Despite these advances, most approaches remain limited to simulation, with experimental demonstrations constrained by the challenges of high‐resolution microfabrication. In particular, achieving pixel‐by‐pixel on‐chip optical regulation remains a critical bottleneck, as ensuring precise alignment, uniformity, and reproducibility across dense µLED arrays is still technically challenging. For AR modules that demand high pixel density, compact footprints, and stringent power efficiency, there is an urgent need for optical regulation strategies that combine high efficiency, strong directionality, and high integrability.

**FIGURE 1:**
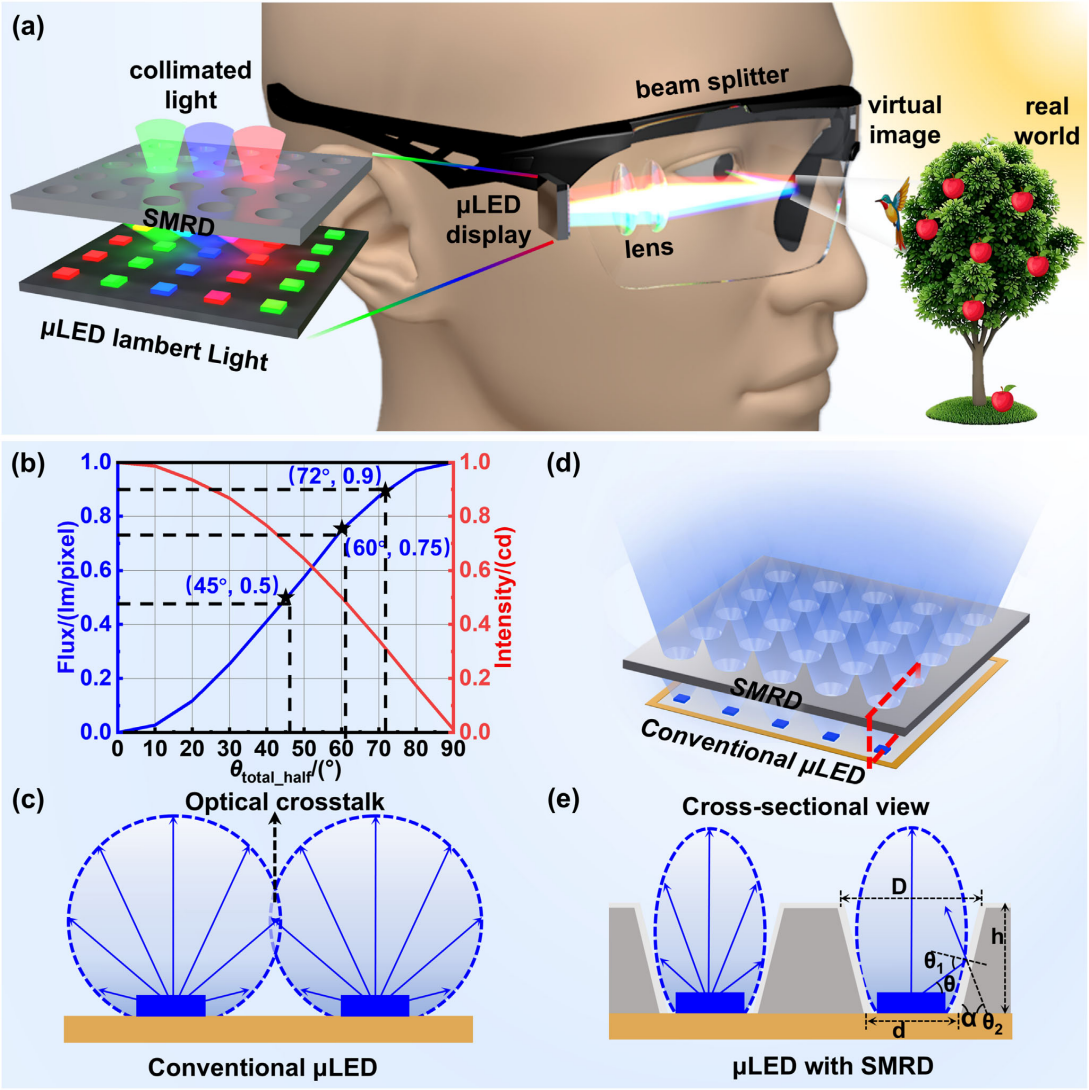
Design process of AR projection optical engine. a) Schematic optical architecture of a direct‐view near‐eye display engine. b) Angular distribution of intensity and flux for a conventional µLED pixel. c) Lambertian emission profile of conventional µLED. d) Schematic of a µLED array integrated with SMRDs. e) Cross‐sectional view and optical path diagram of the µLED/SMRD integrated structure.

Here, we propose and experimentally demonstrate an on‐chip silicon‐based micro‐reflector device (SMRD) that simultaneously enhances light extraction and tailors beam directionality, serving as a core element of direct‐view AR optical projection engines (Figure 1a). The SMRD is fabricated by sculpting microcavity arrays into a monocrystalline silicon substrate and coating the inner sidewalls with a high‐reflectivity silver layer, forming a compact and fabrication‐compatible structure. Comprehensive optical simulations and experimental characterization identify the critical structural parameters and elucidate their synergistic regulation mechanisms. High‐performance on‐chip device fabrication is successfully realized based on the designs. Experimental results confirm that the SMRD effectively redistributes µLED emission, enabling directional beam control and improved light utilization. Importantly, the SMRD enables pixel‐level, one‐to‐one integration with µLED arrays, ensuring precise optical alignment and scalability. Leveraging the silicon platform's intrinsic compatibility with standard semiconductor processes, the device supports wafer‐level fabrication and seamless hybrid integration. Moreover, its on‐chip integrated requires no additional lateral space, preserving array compactness while substantially improving efficiency and directionality. This work establishes a practical and scalable pathway toward high‐efficiency, highly directional, and miniaturized AR projection engines, while also expanding the potential of µLED‐based light‐field modulation in spatial light control and near‐eye display applications.

## 2 Results and Discussion

### 2.1 Design and Simulation

Figure 1b shows the spatial intensity distribution of a conventional µLED pixel and the corresponding flux variation with divergence angle. When the emission half‐angle (*θ_total_half_*) is 45° and 60°, the collected flux accounts for approximately 50% and 75% of the total output, respectively. To capture ∼90% of the total flux, *θ*_total_half_ must be confined to ∼72°. However, as pixel pitch decreases, the inherently wide‐angle emission inevitably induces severe inter‐pixel crosstalk (Figure 1c). At the same time, a substantial portion of the emitted light cannot be efficiently collected, resulting in a pronounced reduction in overall system light‐utilization efficiency.

To overcome these limitations, we propose a pixel‐level conventional µLED (referring specifically to the bare µLED without any packaging) array integrated with SMRDs, as shown in Figure 1d. The device consists of two components: the upper SMRD structure and the lower conventional µLED array, precisely aligned at the chip center. Owing to the beam‐shaping capability of the SMRD at the pixel level, this architecture not only tailors the angular emission profile but also effectively suppresses inter‐pixel crosstalk, as demonstrated in Figure 1e.

To simplify analysis, the point‐source approximation was employed to derive the geometric relationship between the emission angle and the structural parameters (while in the actual simulations, the µLED was modeled using a standard Lambertian light‐source model) [50], as illustrated in Figure 1e:

(1) $$\left\{\begin{array}{c} \theta_{1}=\frac{\pi }{2}+\theta -\alpha \\ \alpha =\arctan\frac{h}{\left(\frac{D-d}{2}\right)}\\ \theta_{2}=\frac{\pi }{2}+\theta_{1}-\alpha \end{array}\right.$$

where, h denotes the SMRD height, while D and d represent the diameters of the top and bottom apertures, respectively. The sidewall tilt angle is defined as *α*, *θ* is the µLED emission angle relative to the horizontal plane, *θ_1_* is the reflection angle on the inclined surface, and *θ_2_* corresponds to the final output direction. The output direction is therefore jointly governed by these geometric parameters, highlighting the importance of rational structural design for efficient beam shaping.

To minimize energy loss and enhance beam collimation, [51, 52] a silver reflective coating was applied to the SMRD sidewalls [53, 54]. Silver exhibits >95% reflectance across the RGB spectral bands (Figure S1a,b), substantially outperforming conventional metals such as aluminum, thus improving both light‐extraction efficiency and overall optical performance [55].

The key design parameters of the SMRD include the height (h), sidewall tilt angle (α), and bottom aperture diameter (d), with α jointly determined by h and d. Increasing the cavity height (*h*) leads to a steeper sidewall and a larger tilt angle, whereas decreasing the bottom aperture (*d*) results in a more gradual sidewall and a smaller tilt angle. We systematically analyzed the effects of varying hx (*x* = 200–800 µm, step 100 µm) and dy (*y* = 390–450 µm, step 10 µm) on µLED light‐extraction efficiency, beam divergence, and pixel crosstalk (details in the Appendix 4.2 and Figure S2). For clarity, hx and dy are used in the following text to denote different heights and bottom aperture diameters, respectively (all dimensions are in µm).

**FIGURE 2:**
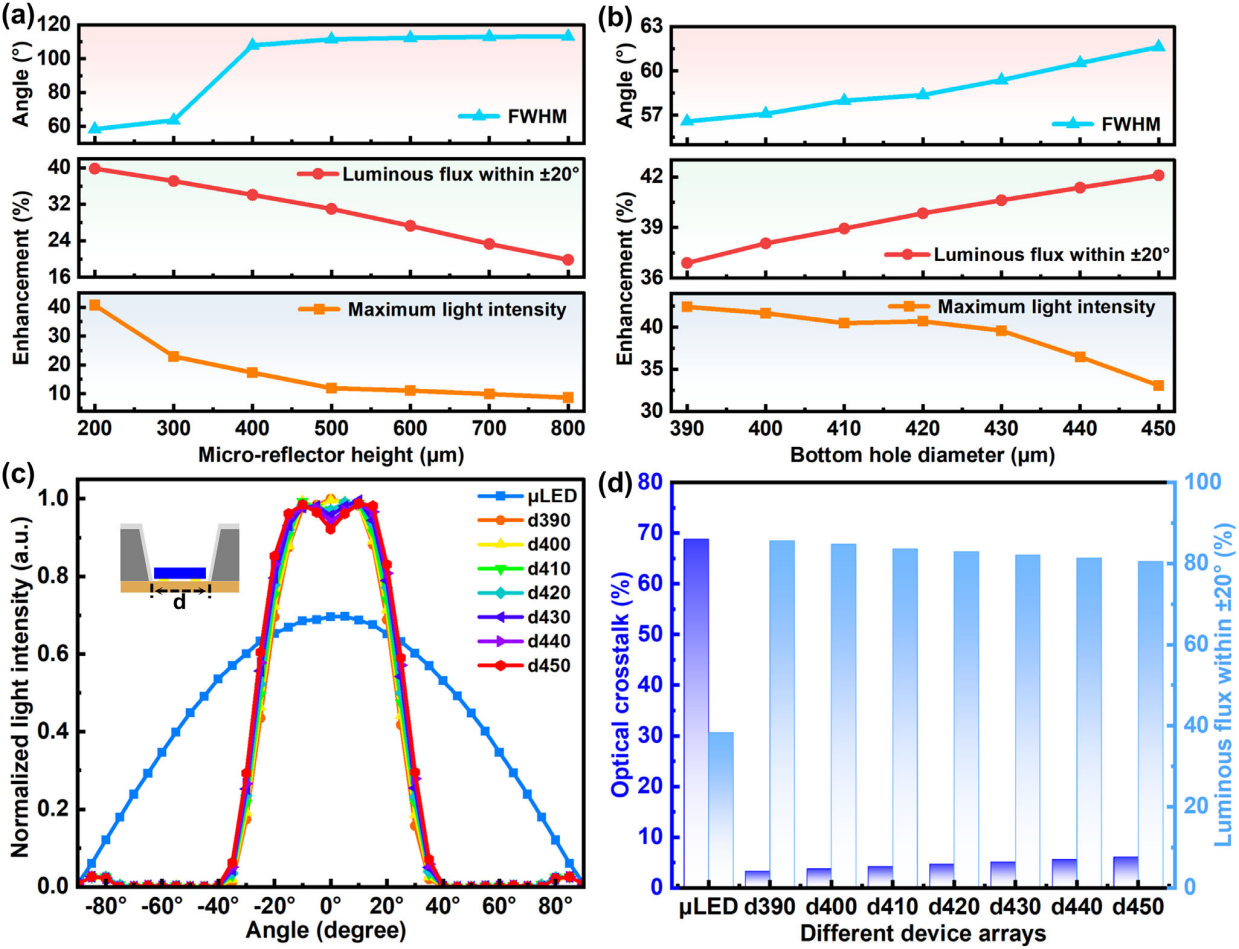
Simulated results of pixel‐level SMRDs with a) varying heights and b) different bottom aperture diameters. c) Normalized intensity distribution of 5 × 5 µLED/SMRD integrated arrays with different bottom apertures. d) Optical crosstalk and luminous flux of 5 × 5 µLED/SMRD integrated arrays as a function of bottom aperture diameter.

As shown in Figure 2a, increasing h reduces central intensity and ±20° luminous flux while broadening the FWHM. The optimal case was observed at h = 200 µm, yielding a 40.68% enhancement in central intensity, a 39.84% increase in ±20° luminous flux, and FWHM compression to ±29.2° (∼2.06× reduction) compared with conventional µLEDs. Further tuning of d (Figure 2b) revealed an optimum at d = 390 µm, resulting in a 42.4% increase in central intensity, a 36.89% rise in ±20° luminous flux, and FWHM compression to ±28.3° (∼2.12× reduction). These results demonstrate that SMRD integration enables simultaneous enhancement of central brightness and optical collection efficiency while effectively tailoring the emission angle, offering a promising pathway for high‐efficiency beam shaping.

Building on the pixel‐level optimization, a 5 × 5 array structure was simulated (modeling parameters are shown in Figure S3). As shown in Figure 2c, the normalized intensity distribution and FWHM of µLED/SMRD integrated arrays with different bottom apertures (d390–450) were compared with conventional µLED arrays. The optimum was achieved at d390, where the central intensity increased by 43.6% and the FWHM was compressed to ±23.6°, corresponding to a 2.54× reduction relative to the conventional one. Moreover, as illustrated in Figure 2d, the luminous flux fraction within ±20° exceeded 50% for all SMRD‐integrated arrays, confirming the significant advantage of SMRDs in enhancing both light‐extraction efficiency and emission directivity.

In addition to brightness and directivity, SMRDs exhibit excellent performance in suppressing inter‐pixel crosstalk. To quantitatively evaluate this effect, the optical crosstalk between adjacent µLED pixels was defined η_*Oc*_, and calculated according to Equation (2) [56]:

(2) $$\eta_{\mathrm{Oc}}=\frac{I_{leakage}}{I_{total}}=\frac{I_{R_{1}}+\cdots +I_{R_{n}}}{I_{total}}$$

where *I_total_* denotes the total intensity collected by a detector above the entire array, while *I_leakage_* represents the leakage intensity received by neighboring pixels when only one pixel is turned on. In the simulation, the detector for *R_n_* was positioned above the non‐emitting neighboring pixels, such that *I_leakage_* corresponds to the sum of signals received by surrounding pixels. As shown in Figure 2d (left axis), the conventional µLED array exhibits a crosstalk value as high as 68.8%, whereas the SMRD‐integrated array reduces the maximum crosstalk to only 6.1%. These results clearly demonstrate that the proposed SMRD structure effectively suppresses lateral light leakage, thereby enhancing contrast and image sharpness in µLED arrays (simulation details and crosstalk distribution are shown in Figure S4).

### 2.2 Preparation and Characterization

**FIGURE 3:**
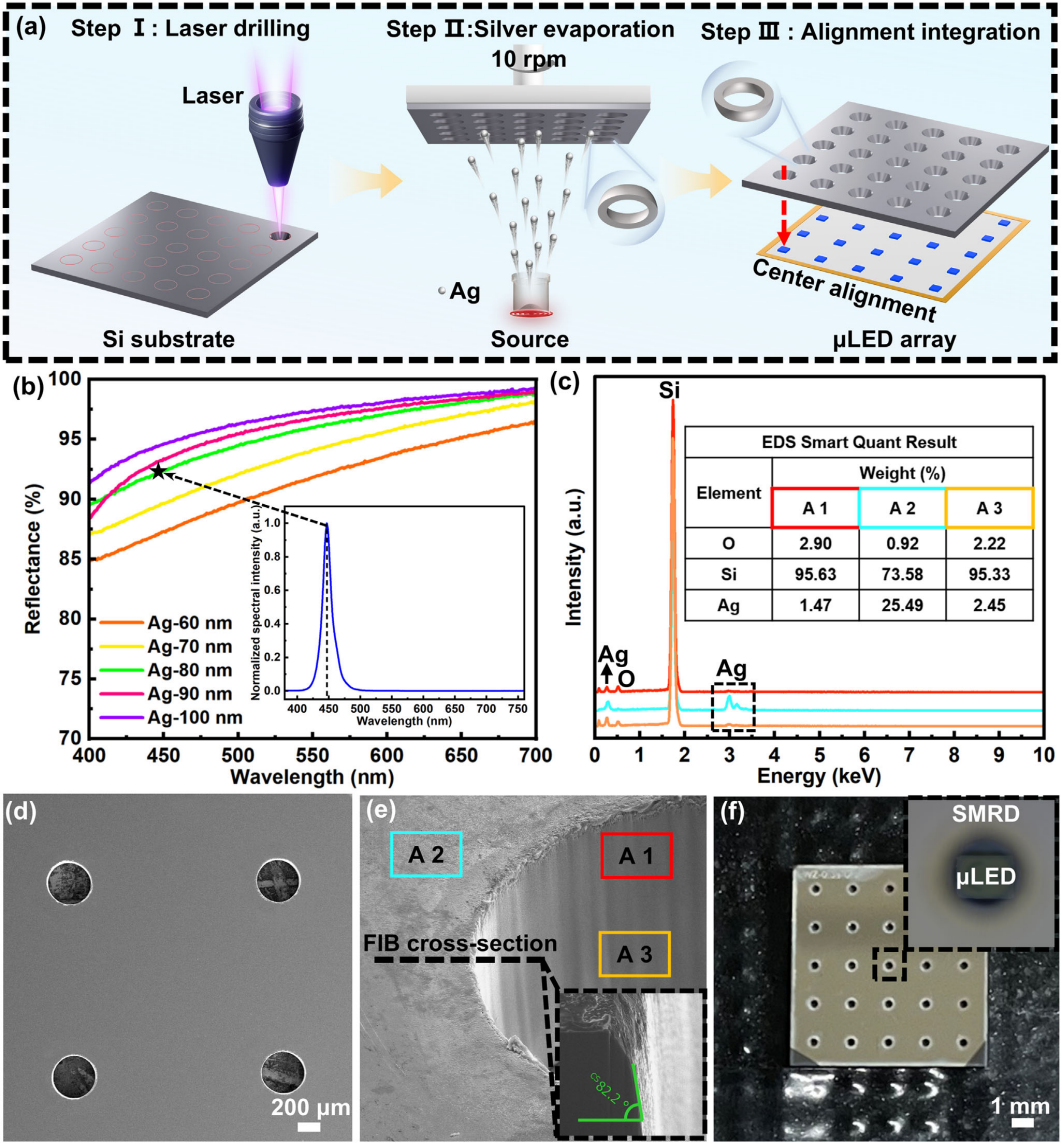
Preparation and characterization process. a) Schematic illustration of the SMRD's fabrication process. b) Reflectivity of Ag films with different thicknesses in the visible spectrum (the dotted line in the illustration: emission wavelength of the µLED). c) EDS spectrum of the SMRD. d) Local SEM morphology of the SMRD. e) FIB cross‐sectional image of a single SMRD. f) Schematic illustration of the on‐chip integrated µLED/SMRD structure.

Based on the preceding Monte Carlo ray‐tracing simulations, SMRDs with a fixed height of h200 and bottom aperture diameters of d390, d400, d410, and d420 were fabricated via picosecond laser micromachining, followed by thermal evaporation coating (Figure 3a). The detailed fabrication procedure is provided in Appendix 4.1. Beyond geometric parameters, SMRD performance critically depends on achieving a high‐reflectivity silver layer on the inner surface. To optimize the reflector thickness, silver films with thicknesses ranging from 60 to 100 nm (10 nm increments) were deposited on clean silicon wafers, and their reflectance in the visible spectrum was measured (Figure 3b). Considering the blue µLED's peak emission wavelength (≈446 nm, inset of Figure 3b), an 80 nm silver film—exhibiting >90% reflectance at this wavelength—was selected as the reflector to ensure efficient light extraction.

Taking the *d*390 SMRD as an example, energy‐dispersive X‐ray spectroscopy (EDS) was employed to verify the elemental composition of the reflective coating. As shown in Figure 3c, the EDS mapping clearly reveals a uniform distribution of Ag along the inner surface of the Si cavity, confirming the successful formation of the silver reflective layer. Figure 3d shows the scanning electron microscopy (SEM) morphology. The results confirm a uniform and intact morphology, with the silver film continuously covering the entire inclined sidewall, thereby verifying the stability and controllability of the fabrication process. Cross‐sectional imaging using focused ion beam (FIB) microscopy (Figure 3e) reveals a sidewall tilt angle of 82.2°, closely matching the design value and indicating excellent processing precision (Figure S5).

Figure 3f shows the on‐chip integrated blue µLED/SMRD device, where precise alignment between the conventional µLED pixel center and the SMRD is clearly observed under optical microscopy. These results demonstrate that the fabrication process ensures structural uniformity and provides a reliable experimental foundation for subsequent brightness measurements and array‐level performance evaluation (Conventional µLED chip and array parameters are shown in Figure S6).

### 2.3 Optical Performance

**FIGURE 4:**
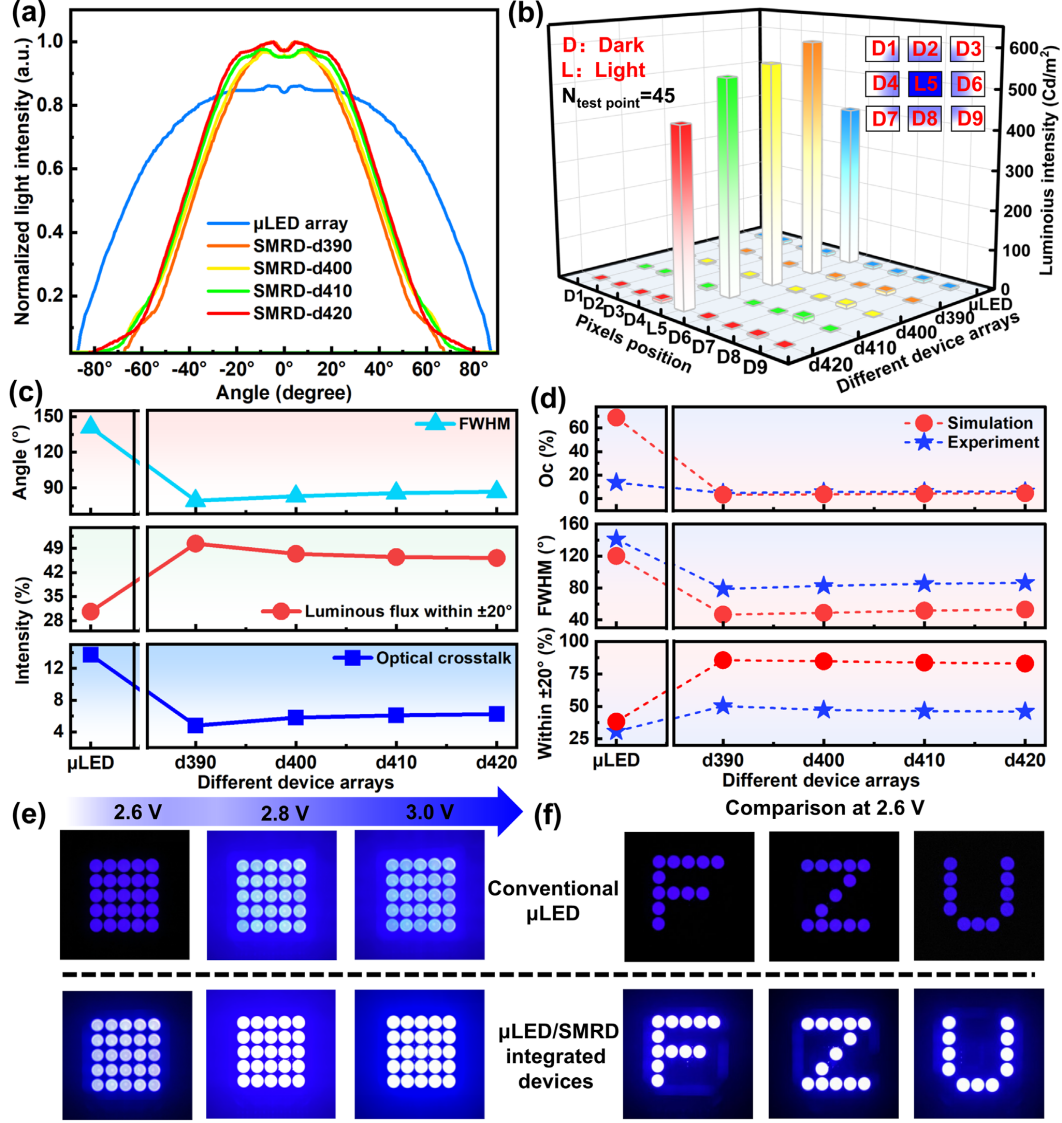
The optical performance integrated by SMRD. a) Emission profiles of conventional µLEDs and µLED/SMRD integrated devices with different bottom aperture diameters. b) Crosstalk measurements of adjacent pixels in conventional µLEDs and µLED/SMRD integrated devices with different bottom aperture diameters (N_test point_ = 45). c) Comparison of FWHM, optical crosstalk ratio, and luminous flux ratio within ±20° for different device arrays. d) Experimental and simulated results of FWHM, optical crosstalk ratio, and luminous flux ratio within ±20° for different device arrays. e) Brightness comparison between SMRD‐d390 and conventional µLEDs under different driving voltages. f) Direct display of the “FZU” pattern: comparison between SMRD‐d390 and conventional µLEDs at 2.6 V.

Under identical testing conditions (details in the Appendix 4.2), we compared the normalized emission intensities of conventional µLEDs with µLED/SMRD integrated devices varying aperture diameters (*d*390, *d*400, *d*410, *d*420), as shown in Figure 4a. On‐chip SMRD integration significantly narrows the emission profile, imparting strong beam‐collimation characteristics compared with conventional µLEDs (FWHM = ±70.5°). With increasing SMRD aperture size, the emission angle gradually broadens (Figure 4c, top). The *d*390 device achieves the best performance, compressing the FWHM to ±39.4°—nearly 1.8× narrower than that of the conventional µLED. Within the ±20° range, the luminous flux of the *d*390 device increases by up to 64% (Figure 4c, middle), confirming its superior light‐utilization efficiency.

To assess pixel crosstalk suppression, we measured the luminance distribution of adjacent pixels in conventional µLED arrays and SMRD‐integrated arrays when only the central pixel was activated, as shown in Figure 4b (the corresponding statistical results are summarized in Table S1). All SMRD‐integrated arrays exhibited enhanced brightness, with the *d*390 device achieving a peak luminance increase of ∼45.64%. Simultaneously, crosstalk was markedly reduced: while conventional µLED arrays exhibited severe crosstalk, the *d*390 device lowered the crosstalk ratio to 4.75% (Figure 4c, bottom). These results demonstrate that SMRDs not only enable efficient beam shaping but also significantly improve array contrast and image clarity. Furthermore, comparison with existing approaches as shown in Table 1, SMRDs achieve light‐collection efficiency on par with microlens arrays, while offering distinct advantages in crosstalk suppression and fabrication simplicity.

**TABLE 1:** Comparison of current technologies for enhancing the luminous efficiency and beam directionality of µLEDs.

Key technology	Result	Beam shaping	Light‐gathering efficiency	Optical crosstalk	Preparation process	Refs.
Photonic crystal	Theoretical	±30°	65.4%	—	Difficulty	[26]
Metasurface	Theoretical	±30°	52.6%	—	Difficulty	[29]
Resonant cavity	Experimental	±39.4°	—	—	Medium	[57]
Free‐form surface microlens	Theoretical	±20°	86.8%	—	Difficulty	[15]
Micro‐lens array	Experimental	±20°	62.0%	Low	Medium	[27]
SMRD	Experimental	±20°	64.0%	4.75%	Easier	This work

Figure 4d compares experimental results with simulations in terms of crosstalk ratio, FWHM, and luminous flux within ±20° for conventional µLEDs and µLED/SMRD integrated devices with different apertures. The experimental and simulated trends agree well overall, though minor discrepancies remain. The most notable deviation occurs in the crosstalk ratio of conventional µLEDs, primarily due to differences in methodology: experimental measurements employed a luminance meter with a 1° field of view, whereas simulations used far‐field receivers, leading to variations in light collection and spatial sampling.

To further validate SMRD performance in practical applications, we compared conventional µLEDs with the best‐performing d390 µLED/SMRD integrated device in a 5 × 5 array under drive voltages of 2.6, 2.8, and 3.0 V (Figure 4e). In all cases, the SMRD‐integrated arrays exhibited superior brightness, while the apparently limited visual improvement in halo suppression was mainly attributed to the restricted dynamic range of the imaging system and residual optical‐path scattering, rather than the intrinsic performance of the SMRD. Notably, at 2.6 V, when displaying the “FZU” pattern, the µLED/SMRD integrated array delivered significantly higher brightness and contrast compared with the conventional µLED array (Figure 4f). Consequently, subsequent AR display experiments in this study were conducted using *d*390 µLED/SMRD integrated devices.

### 2.4 AR Display Application

**FIGURE 5:**
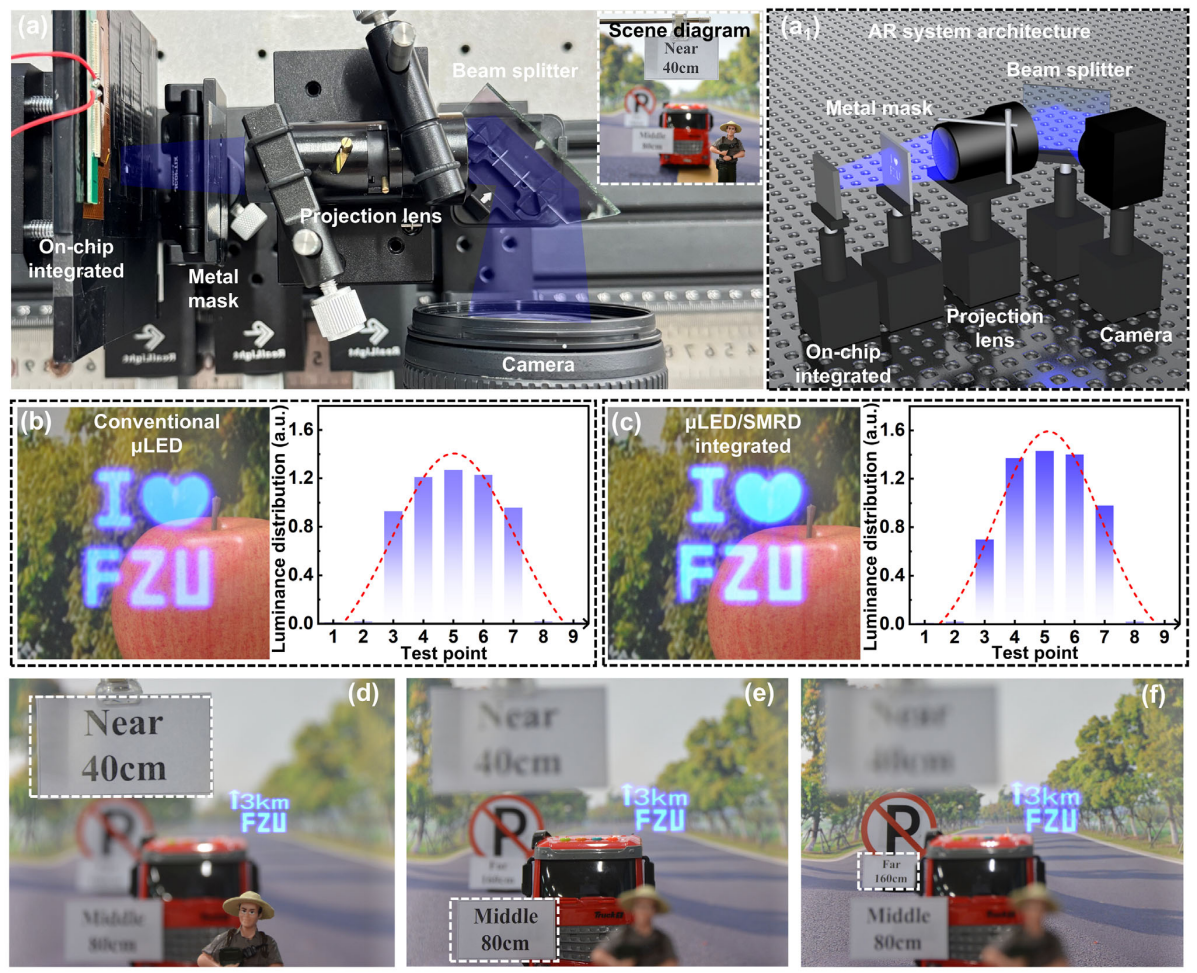
AR display system architecture and applications. a) Experimental setup of the AR near‐eye display prototype, including distance reference objects positioned at 40, 80, and 160 cm [a_1_) AR system architecture]. b, c) AR display uniformity of the prototype equipped with b) the conventional µLED and c) the µLED/SMRD integrated device. d–f) Demonstration of AR display results with the µLED/SMRD‐d390 at viewing distances of 40–160 cm.

As shown in Figure 5a, an AR near‐eye optical prototype was constructed to experimentally validate the proposed device (the AR system architecture as shown in Figure 5a1, which corresponds to the experimental setup). The system was mounted on an optical bench and comprised a µLED/SMRD integrated device (*d*390), a metal mask, a projection lens, a beam splitter, and a camera, with the camera serving as an eye substitute to record imaging results. To provide spatial references, distance markers and model objects were placed at 40, 80, and 160 cm along the optical path (inset, upper right).

Building on the earlier device‐level analysis, we systematically compared the projection performance of conventional µLEDs and µLED/SMRD integrated devices (*d*390) within this prototype. Figure 5b,c presents the emission intensity distributions and imaging outcomes under identical driving conditions. SMRD‐integrated µLEDs delivered substantially enhanced projection brightness and optical efficiency, thereby exhibiting superior source characteristics for near‐eye display architectures.

The practical AR projection performance of blue µLED/SMRDs integrated device is further demonstrated in Figure 5d–f. When the reference objects were shifted from 40 to 160 cm, the clarity of the virtual image remained consistently stable, verifying the robustness of the optical configuration for spatial imaging (Video S1). Moreover, analogous experiments with green and red µLED/SMRDs integrated device (Figure S7) confirm the scalability of this strategy toward full‐color AR display applications.

Finally, the demonstrated AR prototype highlights the potential of the SMRD‐enabled µLED platform for high‐efficiency near‐eye displays, while further scaling to smaller pixel dimensions and advanced fabrication strategies is discussed in the Supporting Information as a direction for future work (Figure S8).

## 3 Conclusion

In this work, we propose and experimentally demonstrate an on‐chip SMRD that simultaneously enhances light extraction and shapes emission, with its effectiveness in µLED light‐field modulation validated through both simulations and experiments. The SMRD markedly improves µLED emission characteristics, reducing the FWHM from ±70.5° to ±39.4°, increasing luminous flux within ±20° by ∼64%, and suppressing pixel crosstalk to 4.75%. Beyond device‐level performance, integration into a prototype AR display system results in substantially higher image brightness, lower crosstalk, and improved visual clarity across viewing distances of 40–160 cm, confirming its practical potential for near‐eye applications. Beyond its optical performance, the SMRD architecture inherently supports one‐to‐one pixel‐level integration with µLED arrays, enabling precise spatial light regulation at the chip scale. Owing to its silicon substrate, the device demonstrates excellent compatibility with standard semiconductor fabrication processes, enabling direct on‐chip integration. This structural simplicity and process adaptability make the SMRD highly scalable for dense array configurations and large‐scale manufacturing of AR display modules. Overall, this work presents a practical and fabrication‐compatible strategy for realizing efficient, directional, and miniaturized µLED projection systems, paving the way for advanced applications in spatial light modulation, near‐eye displays, and integrated photonic architectures.

## 4 Appendix: Materials and Methods

### 4.1 Fabrication of SMRD‐Integrated µLED Arrays

Micro‐pixel array devices were fabricated on (100)‐oriented single‐crystal silicon substrates. High‐precision direct laser writing was performed at predefined pixel locations using a custom 355 nm picosecond laser system. This process generated localized silicon ejection, producing micro‐particles on surfaces and sidewalls. To improve surface planarity and cleanliness, anisotropic wet etching was carried out in a solution of 25.5% KOH and 5.7% IPA at 60°C for 30–50 min. After etching, the samples were thoroughly cleaned and dried. The silicon microarrays were then inverted and mounted in a high‐vacuum thermal evaporation system (SPECTROD 150, Kurt J. Lesker Company), rotated at 10 RPM, and coated with a uniform silver reflective layer. Film thickness was precisely monitored using a quartz crystal oscillator to meet optical design requirements. Finally, under optical microscope guidance, the SMRDs were accurately aligned and integrated with a blue µLED array (λ = 446 nm), completing the functional integration of µLED/SMRD devices (Detailed alignment and integration steps are provided in Figure S9).

### 4.2 Characterization

To comprehensively evaluate the structural features and optical performance of the fabricated SMRD arrays, multiple characterization techniques were employed. Device surface and sidewall morphologies were examined using an optical microscope (M360‐HK820), a field‐emission scanning electron microscope (FE‐SEM, Nova NanoSEM 230), and a focused ion beam SEM (FIB‐SEM, Helios G4 CX). Material composition and reflective layer quality were analyzed via energy‐dispersive X‐ray spectroscopy (EDS) and a UV–Vis–NIR spectrophotometer (Cary 7000) to determine elemental distribution and wavelength‐dependent reflectivity.

### 4.3 Simulation Design

To systematically investigate the influence of silicon micro‐reflector structural parameters on µLED beam‐shaping performance, 3D Monte Carlo ray‐tracing simulations were conducted. The simulations evaluated the effects of SMRD integration on brightness enhancement, emission‐angle distribution [characterized by full width at half maximum (FWHM)], and pixel crosstalk suppression in conventional µLEDs.

The model incorporated fabrication feasibility: each µLED emitter was defined with dimensions of 300 µm × 220 µm × 100 µm, and array pitches were set to 1874 µm (X‐axis) and 1780 µm (Y‐axis). The initial SMRD height was 500 µm, with a bottom aperture of 420 µm and a top aperture designed according to a 1.13:1 ratio. Therefore, before constructing the 3D structure model, we had already calculated the target design inclination angle (82.875°) based on the above geometric parameters and Equation (1) in the revised version, and accordingly established the 3D SMRD structure for the simulation. Device sidewalls were coated with a high‐reflectivity silver layer. Thin‐film simulations indicated that silver layers thicker than 60 nm achieve reflectivity exceeding 90%, effectively enhancing beam collimation and optical coupling efficiency. Through systematic variation of geometric and optical parameters, the simulations provided theoretical guidance for optimizing SMRD structures, laying the foundation for high‐efficiency, highly directional µLED light engines.

### 4.4 Statistical Analysis

To quantitatively evaluate the impact of SMRD integration on optical crosstalk suppression, a spectroradiometer (SRC‐200 M) with a field of view of 1° was employed. The measurement distance was fixed at 80 cm, and the optical intensity of each individual pixel region was recorded. In this experiment, both a conventional 5 × 5 µLED array and an SMRD‐integrated µLED array were measured, with only the central pixel activated. A total of 45 intensity values were collected. The crosstalk levels of surrounding pixels were calculated and analyzed according to Equation (2). The processed data were subsequently imported into Origin 2022 to generate the crosstalk comparison statistics shown in Figure 4b.

To quantify the enhancement in luminous intensity induced by SMRD integration, a goniophotometer (GO‐i300) was used to measure the angular emission intensity distributions of µLED arrays before and after SMRD integration. Measurements were conducted on five independent device arrays for each configuration. The relative improvement in luminous intensity was calculated using Equation (3):

(3) $$\eta_{I}=\frac{I_{SMRD}-I_{\mu LED}}{I_{\mu LED}}\times 100\%$$

where *I_SMRD_* and *I*_µ*LED*_ represent the measured emission intensities of the conventional µLED array and with SMRD integration, respectively. The calculated results were imported into Origin 2022 to generate the emission intensity and angular distribution curves presented in Figure 4a.

## Author Contributions

W.L. was associated with conceptualization, investigation, experiments, simulations, formal analysis, writing – original draft and review and editing, methodology, and resources. J.C., Z.F., Y.C., Z.Y., and S.Y. were associated with methodology and data curation. Y.Y., S.X., Q.Y., and T.G. was associated with formal analysis, funding acquisition, writing – review and editing, and resources. E.C. was associated with conceptualization, formal analysis, writing – review and editing, resources, and project administration supervision. All authors analyzed and discussed the results.

## Conflicts of Interest

The authors declare no conflicts of interest.

## Supporting information




**Supporting File 1**: advs73726‐sup‐0001‐SuppMat.docx.

**Supporting File 2**: advs73726‐sup‐0002‐VideoS1.mp4.

## Data Availability

The data that support the findings of this study are available from the corresponding author upon reasonable request.
