# Supplementary material for: On‐Chip Silicon‐Based Micro‐Reflector Pixelated with Micro‐Light‐Emitting Diodes for Augmented Reality Projection Displays
Source: Adv Sci (Weinh). 2026 Jan 7;13(16):e23254. doi: 10.1002/advs.202523254 (PMC13042926; doi:10.1002/advs.202523254)
Supplement: Supplementary file 1 — Supporting File 1: advs73726‐sup‐0001‐SuppMat.docx. [file ADVS-13-e23254-s001.docx]

Supporting Information for: “On-Chip Silicon-Based Micro-Reflector Pixelated with Micro-Light-Emitting Diodes for Augmented Reality Projection Displays”

**Wenzong Lai,^a^ Junhu Cai,^a^ Zhengui Fan,^a^ Yu Chen,^a^ Ziming Yao,^a^ Shuzhan Yan,^a^ Yun Ye,^a,b^ Sheng Xu,^a,b^ Qun Yan,^a,b^ Tailiang Guo,^a,b^ and Enguo Chen^a,b,^***

*^a^National and Local United Engineering Laboratory of Flat Panel Display Technology, College of Physics and Information Engineering, Fuzhou University, Fuzhou, Fujian 350108, P. R. China.*

*^b^Fujian Science & Technology Innovation Laboratory for Optoelectronic Information of China, Fuzhou, Fujian 350108, P. R. China.*

*Enguo Chen, ceg@fzu.edu.cn


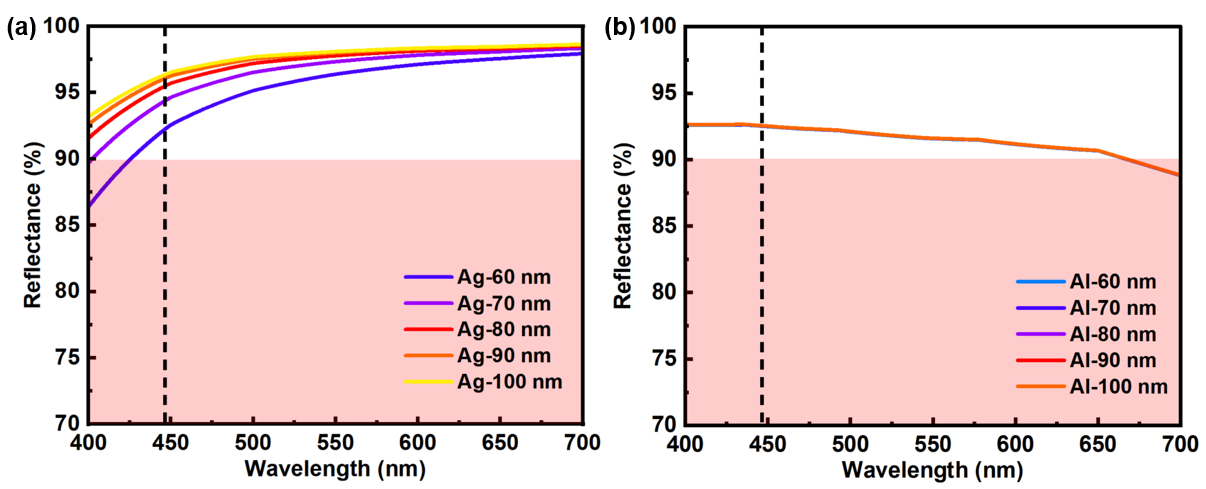


**Fig. S1.** The reflectivity of the 60-100nm Ag reflective layer and Al reflective layer designed by the optical film design software.


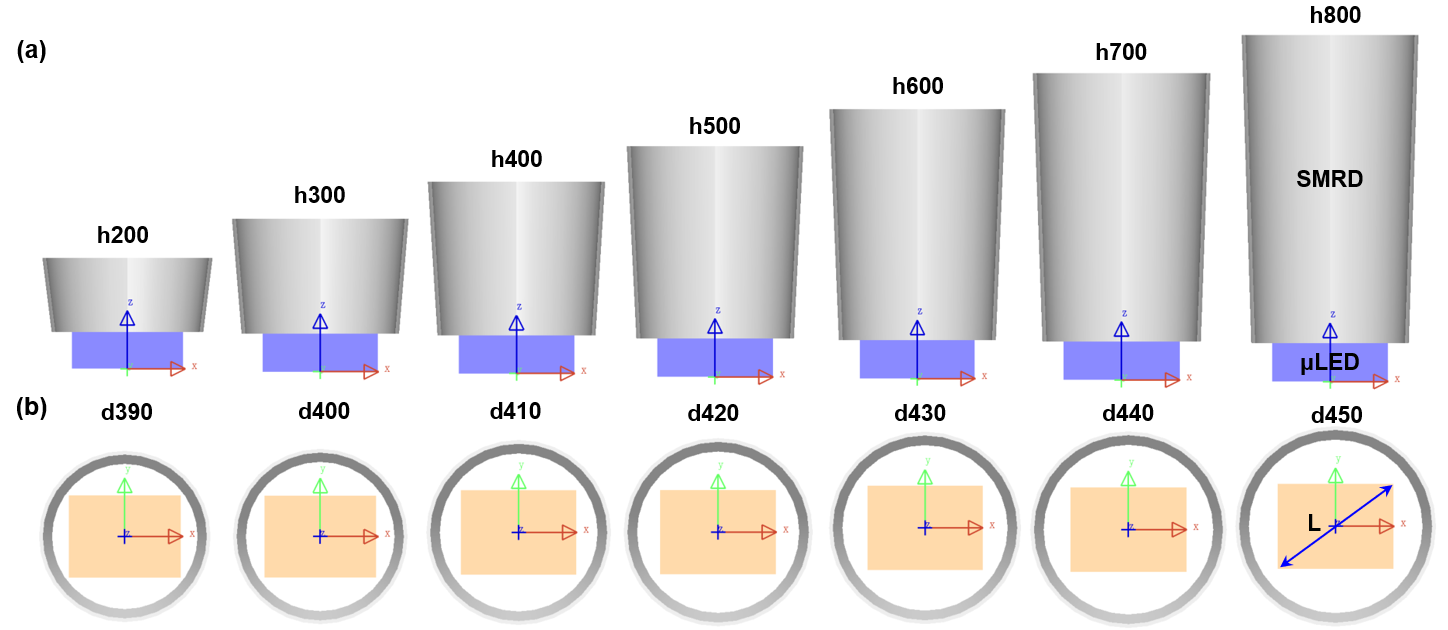


**Fig. S2.** Monte Carlo ray tracing combined with SolidWorks is used to conduct optical modeling of the height and bottom aperture of single SMRD.


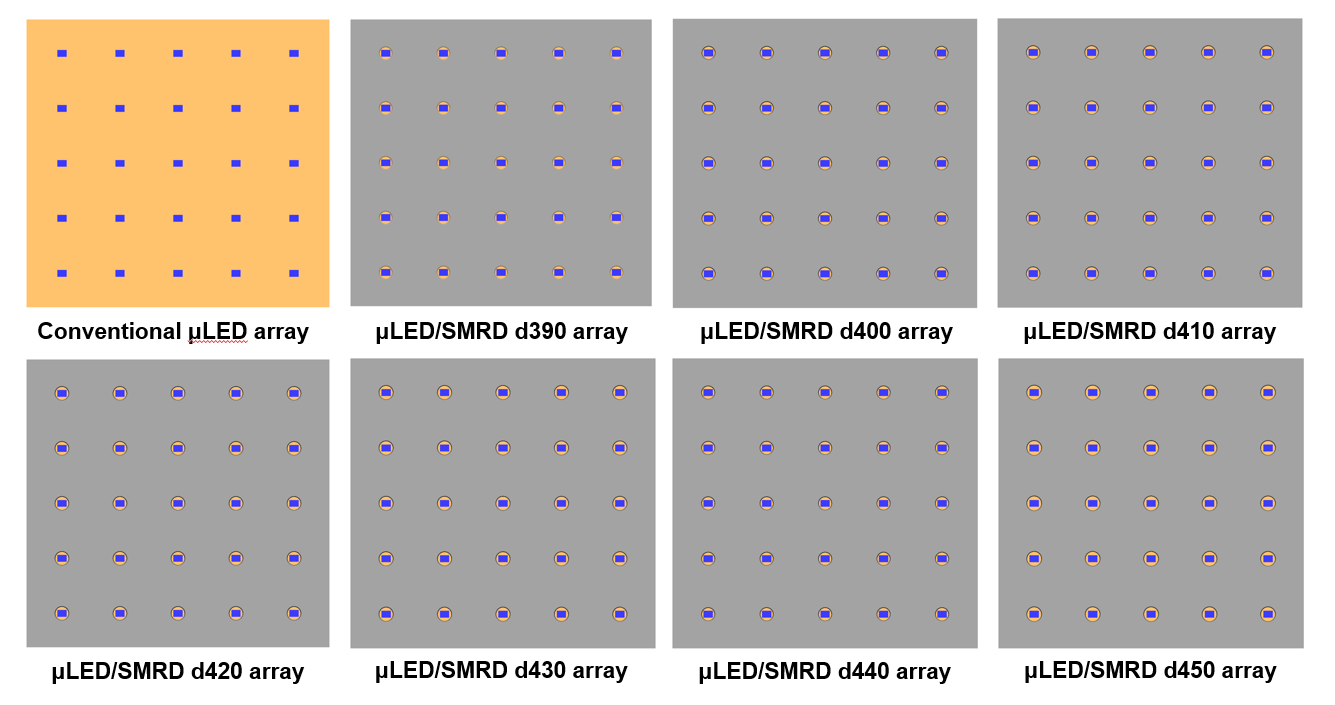


**Fig. S3.** The Monte Carlo ray-tracing method was used to simulate the conventional μLED and the SMRD arrays with a height of 200 μm and different bottom opening diameters.


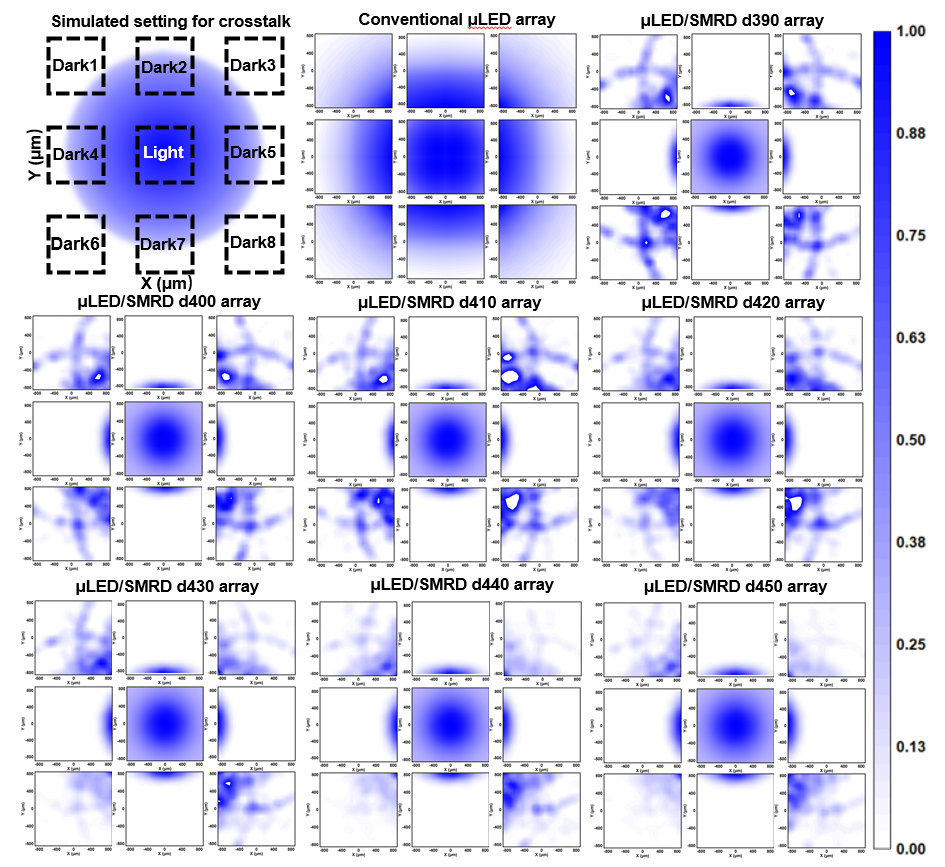


**Fig. S4.** The crosstalk settings during the illumination of the center pixels of conventional μLED and the integrated SMRD arrays with different bottom hole diameters, as well as the intensity distribution of the surrounding pixels.


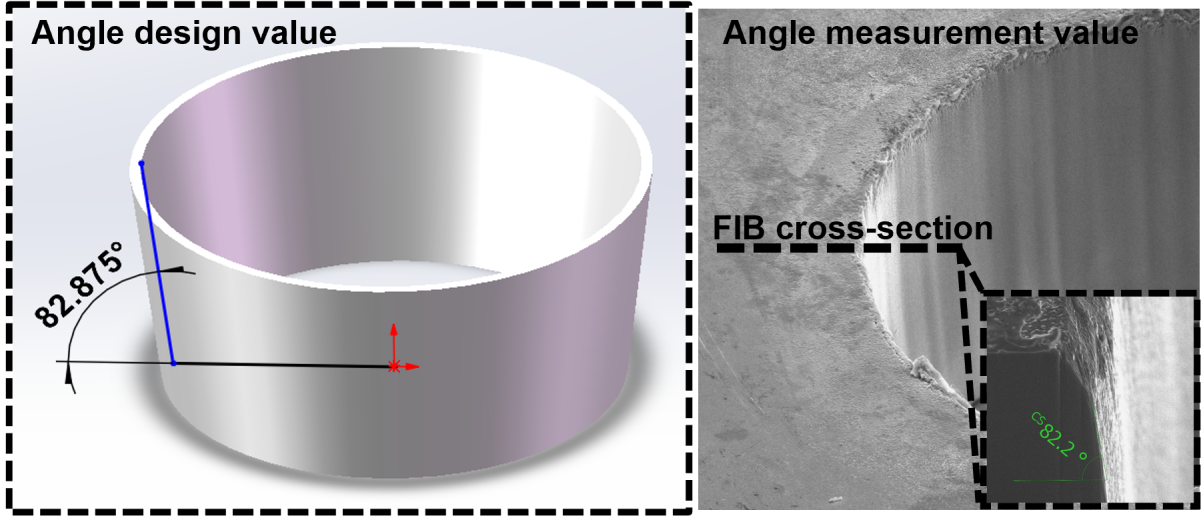


**Fig. S5**. Comparison of the design angle values of SMRD with the measured values from the FIB experiment.


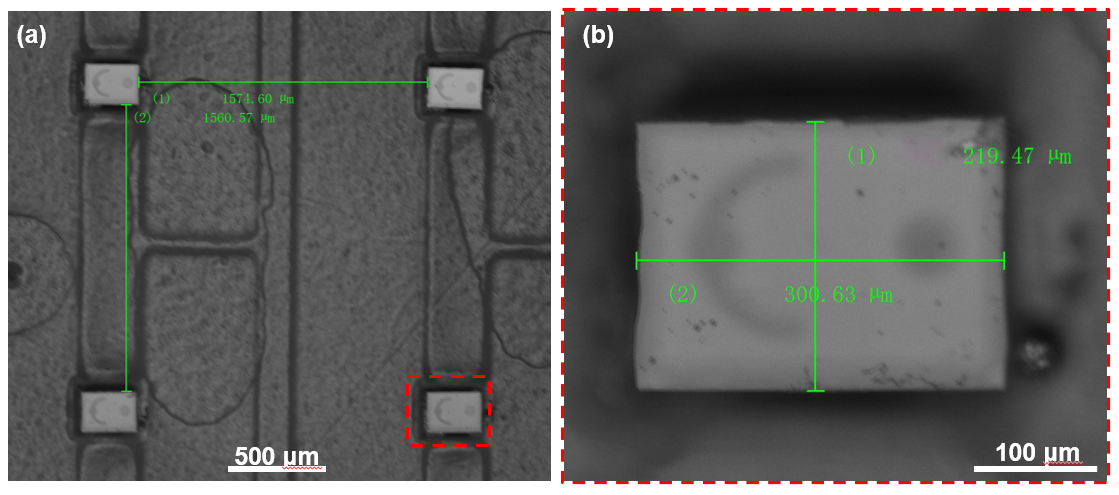


**Fig. S6.** The inter-pixel spacing of the conventional μLED array and the size of a single μLED chip.

**Table S1.** The measurement values of crosstalk between adjacent pixels in conventional μLED with different bottom opening diameters and μLED/SMRD integrated devices.

| Luminoius intensity (Cd/m^2^) | D1 | D2 | D3 | D4 | L5 | D6 | D7 | D8 | D9 |
| --- | --- | --- | --- | --- | --- | --- | --- | --- | --- |
| μLED | 6.57 | 8.94 | 4.55 | 8.68 | 412.57 | 7.68 | 6.89 | 7.90 | 5.30 |
| d390 | 1.91 | 4.08 | 1.47 | 3.17 | 600.85 | 3.34 | 2.43 | 10.7 | 1.47 |
| d400 | 2.23 | 4.53 | 2.19 | 4.75 | 558.84 | 3.30 | 3.87 | 9.81 | 1.41 |
| d410 | 1.15 | 5.98 | 1.43 | 5.64 | 538.84 | 1.18 | 1.69 | 13.8 | 1.63 |
| d420 | 1.35 | 3.48 | 2.23 | 8.97 | 444.41 | 3.25 | 1.39 | 5.48 | 1.35 |


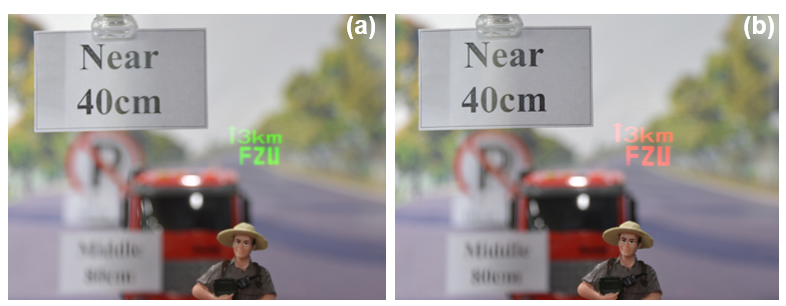


**Fig. S7.** Display performance under green and red wavelengths in the AR display system architecture.


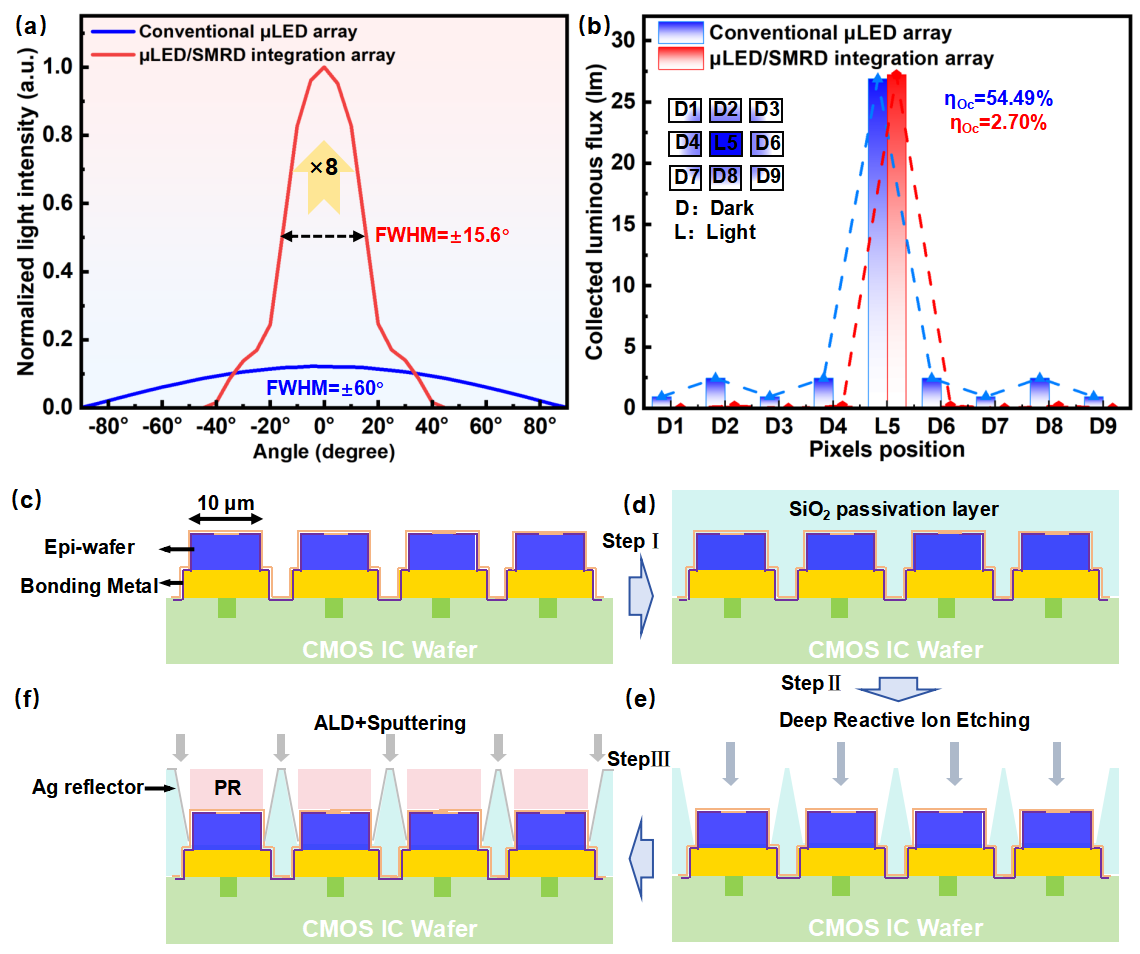


**Fig. S8**. (a) The emission characteristic diagrams of conventional μLED and μLED/SMRD integrated devices when the pixel size is 10 μm. (b) Simulation data of crosstalk between adjacent pixels in conventional μLED and μLED/SMRD integrated devices when the pixel size is 10 μm. (c-f) Schematic illustration of the SMRD fabrication process specifically designed for 10 μm μLED pixels.

As a future research direction, the scalability of the proposed SMRD architecture toward sub-10 μm μLED pixel sizes will be further explored to meet the requirements of high-density AR display applications.

First, it should be emphasized that the core optical mechanism of the SMRD—namely, pixel-level directional confinement and beam shaping enabled by micro-reflective cavities—is, in principle, not dependent on the absolute pixel size. Therefore, as long as the same design concept and geometric scaling relationships are preserved, the SMRD architecture can be conceptually extended to smaller pixel dimensions.

To verify this scalability, we further performed supplementary simulations on 10 μm-scale μLED pixel arrays integrated with SMRD structures. As shown in the simulation results in **Figs. S8(a)** and **(b)**, under preserved relative cavity geometry and reflective sidewall configurations, the SMRD-integrated 10 μm μLED array still exhibits a pronounced optical enhancement, including an approximately eightfold increase in on-axis intensity, a reduced emission divergence of ±15.6°, and suppressed lateral leakage down to 2.7%. Compared with the bare μLED array, the SMRD-integrated design demonstrates a more concentrated beam profile and significantly reduced pixel-to-pixel crosstalk. These results provide design-level validation that the proposed SMRD architecture is scalable to 10 μm pixel dimensions and is not limited to the hundred-micrometer regime.

Meanwhile, it must be emphasized that when the pixel size is scaled down from the hundred-micrometer regime to 10 μm or even smaller dimensions, device integration faces a series of significantly increased fabrication challenges. These limitations do not originate from a breakdown of the optical principles, but mainly arise from manufacturing precision, alignment strategies, and material processing constraints.

To address these scaling requirements, we have added a schematic illustration of the SMRD fabrication process specifically designed for 10 μm μLED pixels, as shown in **Fig. S8(c-f)**. This process adopts a wafer-level, alignment-free monolithic integration strategy, in which the SMRD is fabricated directly on the μLED array via lithographic patterning, thereby eliminating the need for post-alignment steps. The key process steps and considerations are described as follows:

**Step Ⅰ: Deposition of a SiO₂ passivation layer.**

At the 10 μm pixel scale, surface planarity and electrical isolation are critical for achieving uniform microcavity structures. A SiO₂ passivation layer is first deposited on the μLED array to provide inter-pixel electrical isolation, surface planarization, and a functional layer for subsequent microcavity etching, as shown in **Fig. S8(d)**. This step can be realized using mature PECVD or ALD processes ^[i-iii]^, ensuring excellent thickness uniformity across the wafer and providing a stable foundation for precise microcavity definition.

**Step Ⅱ: Microcavity patterning and etching.**

On the SiO₂ passivation layer, SMRD microcavities are patterned on a pixel-by-pixel basis using high-resolution lithography, followed by anisotropic etching to form the reflective cavity structures. At a μLED scale of 10 micrometers, the corresponding SMRD sidewall angle accuracy, cavity depth control, and surface roughness have a pronounced impact on beam-shaping performance. Therefore, the proposed process emphasizes the use of micro-/nano-scale-compatible etching techniques, such as high-precision lithography combined with deep reactive ion etching (DRIE) ^[iv-vii]^, as shown in **Fig. S8(e)**, to ensure high uniformity and reproducibility of cavity geometry across the array.

**Step Ⅲ: Metal deposition on the reflective sidewalls.**

After the microcavities are formed, a metallic reflective layer is deposited on the cavity sidewalls to enable directional confinement of the emitted light. When the cavity dimensions shrink to the μLED-relevant scale (~10 μm), the associated high-aspect-ratio geometries pose significant challenges to achieving conformal metal coating, thereby imposing stringent requirements on deposition techniques. Accordingly, highly conformal deposition strategies are adopted, such as atomic layer deposition (ALD) as a seed layer followed by metal thickening ^[viii, ix]^, as shown in **Fig. S8(f)**, or optimized deposition angles and process parameters to mitigate shadowing effects and ensure uniform sidewall reflectivity, thereby maintaining stable beam-shaping performance.

Through this wafer-level, monolithic SMRD-on-μLED fabrication flow, the integration challenges associated with sub-micrometer alignment tolerances can be effectively avoided, while providing a practical pathway toward high-density integration at the 10 μm pixel scale. Combined with the supplementary simulation results, we believe that, with appropriate micro-/nano-fabrication and thin-film deposition techniques, the proposed SMRD architecture retains clear scalability and application potential for 10 μm μLED pixels.

**References:**

[i] Y. Deng, J. Chen, S. Li, H. Huang, Z. Liu, Z. Yan, S. Lai, L. Zheng, T. Yang, Z. Chen, T. Wu, The impacts of SiO_2_ atomic-layer-deposited passivation layer thickness on GaN-based green micro-LEDs, *Semicond. Sci. Tech.* **2024**, 39, 045004.

[ii] H. Huang, S. Huang, Y. Tsai, S. Wang, Y. Lee, S. Weng, H. Ku, C. Lin, Investigation on reliability of red micro-light emitting diodes with atomic layer deposition passivation layers, *Opt. Express*, **2020**, 28, 38184.

[iii] S. Wong, D. Hwang, A. Alhassan, C. Lee, R. Ley, S. Nakamura, S. DenBaars, High efficiency of III-nitride micro-light-emitting diodes by sidewall passivation using atomic layer deposition, *Opt. express*, **2018**, 26, 21324.

[iv] J. Ai, Q. Du, Z. Qin, J. Liu, X. Zeng, Laser direct-writing lithography equipment system for rapid and μm-precision fabrication on curved surfaces with large sag heights, *Opt. Express*, **2018**, 26, 20965.

[v] M. Hooda, M. Wadhwa, S. Verma, M. Nayak, P. George, A. Paul, A systematic study of DRIE process for high aspect ratio microstructuring, *Vacuum*, **2010**, 84, 1142.

[vi] M. Huff, Recent advances in reactive ion etching and applications of high-aspect-ratio microfabrication, *Micromachines*, **2021**, 12, 991.

[vii] T. Kim, J. Lee, Optimization of deep reactive ion etching for microscale silicon hole arrays with high aspect ratio, *Micro Nano Systems Lett.* **2022**, 10, 12.

[viii] P. Bulkin, S. Gaiaschi, P. Chapon, D. Daineka, N. Kundikova, Protective coatings for front surface silver mirrors by atomic layer deposition, *Opt. express*, **2020**, 28, 15753.

[ix] V. Beladiya, T. Faraz, P. Schmitt, A. Munser, S. Schröder, S. Riese, C. Muehling, D. Schachtler, F. Steger, R. Botha, F. Otto, T. Fritz, C. Van Helvoirt, W. Kessels, H. Gargouri, A. Szeghalmi, Plasma-enhanced atomic layer deposition of HfO_2_ with substrate biasing: thin films for high-reflective mirrors. *ACS appl. Mater. Interfaces*, **2022**, 14, 14677.

**
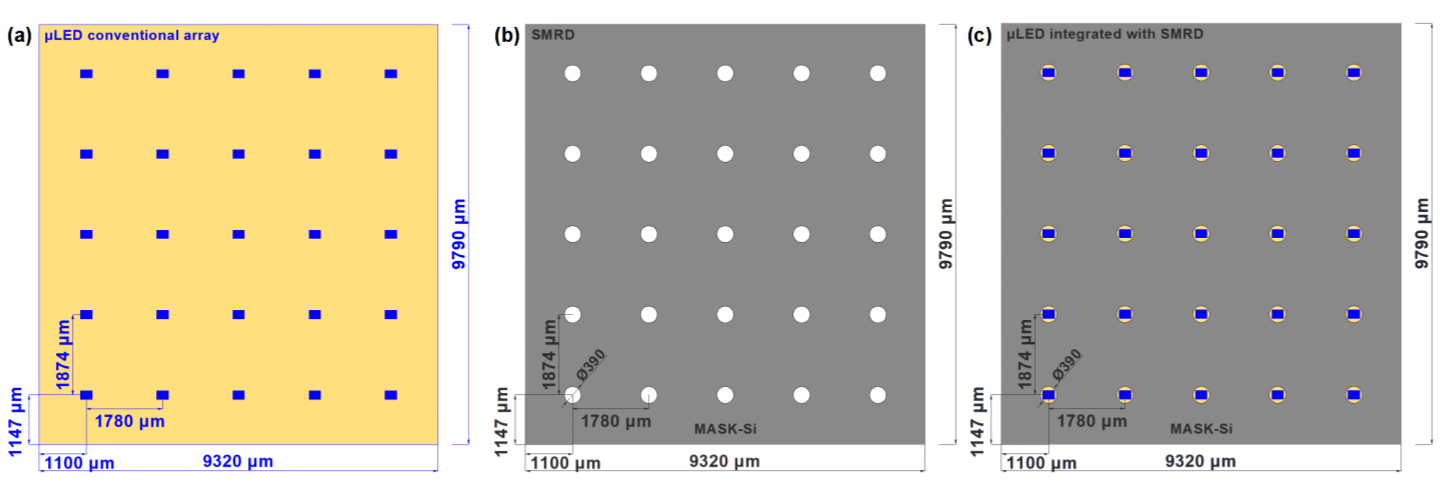
**

**Fig. S9**. (a) The measurement results of the traditional μLED pixel array spacing. (b) The SMRD pixel array mask plate designed according to Figure (a). (c) Schematic diagram of μLED integrated SMRD pixel alignment.

The precise alignment between the SMRD and the μLED array is crucial for achieving pixel-level light regulation, and it mainly involves the following steps:

**1. Alignment Methods**

**Step Ⅰ:** Pixel-matched design and fabrication basis

Prior to SMRD fabrication, we precisely designed the SMRD pixel structures according to the actual pixel size and pitch of the μLED array and generated a corresponding mask file, as illustrated in **Figure S9**. This mask file was imported into a picosecond-laser etching system for array patterning. The picosecond-laser process provides a ±1.5 μm aperture tolerance and ±2 μm positioning accuracy, combined with a motion-stage repeatability of ±1 μm. These fabrication precisions ensure that the fabricated SMRD array remains highly consistent with the μLED array in both dimension and position, thereby providing a solid foundation for subsequent alignment.

**Step Ⅱ:** Global alignment based on array boundaries

During assembly, we first employed the correspondence between the outer boundary of the SMRD device and the overall boundary of the μLED array as the global reference. Assisted by microscopic visualization, this step allows rapid identification of the initial matching region, effectively narrowing the search range for pixel-level alignment and improving the efficiency and robustness of the overall alignment process.

**Step Ⅲ:** Pixel-level fine alignment

After completing the global alignment, pixel-level adjustment was performed using a 50× optical microscope and a high-precision XYZ positioning stage. The stage offers a motion resolution of approximately 0.5–1 μm, enabling precise matching of individual pixels by comparing the SMRD pixel contours with the μLED mesa boundaries. This ensures accurate one-to-one correspondence between the optical structures.

**2. Alignment Tolerance**

With the above two-level alignment strategy, the lateral alignment tolerance achieved under current experimental conditions is approximately ±3 μm. This precision fully satisfies the optical requirements of the SMRD pixel structure and is compatible with the pixel dimensions and optical design of the μLED array. The achieved tolerance benefits from a combination of accurate design, controlled laser-fabrication precision, global boundary referencing, and pixel-level fine tuning.

**3. Bonding Method**

After alignment, **low-temperature UV-curable adhesive** was used for bonding to avoid displacement caused by thermal expansion during high-temperature processing. UV bonding provides stable mechanical support while maintaining the aligned position. Post-bonding inspection under a microscope confirmed that no noticeable misalignment occurred, ensuring mechanical and optical stability during subsequent device characterization.

Overall, the integration process—consisting of precise pixel-matched design, picosecond-laser microfabrication, dual-reference alignment (global + pixel-level), and low-temperature UV bonding—is based on established microfabrication and micro-assembly techniques and is compatible with existing automated alignment and pick-and-place systems. Therefore, it is well-suited for scaling to larger and higher-density μLED arrays.
